# Genetics and the Individualized Therapy of Vestibular Disorders

**DOI:** 10.3389/fneur.2021.633207

**Published:** 2021-02-05

**Authors:** Christine Mei, Hongsong Dong, Eric Nisenbaum, Torin Thielhelm, Aida Nourbakhsh, Denise Yan, Molly Smeal, Yesha Lundberg, Michael E. Hoffer, Simon Angeli, Fred Telischi, Guohui Nie, Susan H. Blanton, Xuezhong Liu

**Affiliations:** ^1^Department of Otolaryngology, University of Miami, Coral Gables, FL, United States; ^2^Shenzhen Second People's Hospital, Shenzhen, China; ^3^Department of Otolaryngology, Boys Town National Research Hospital, Omaha, NE, United States

**Keywords:** genetics, vestibular disorder, individualized therapy, genomics, Meniere disease, usher, BPV, next generation sequencing

## Abstract

**Background:** Vestibular disorders (VDs) are a clinically divergent group of conditions that stem from pathology at the level of the inner ear, vestibulocochlear nerve, or central vestibular pathway. No etiology can be identified in the majority of patients with VDs. Relatively few families have been reported with VD, and so far, no causative genes have been identified despite the fact that more than 100 genes have been identified for inherited hearing loss. Inherited VDs, similar to deafness, are genetically heterogeneous and follow Mendelian inheritance patterns with all modes of transmission, as well as multifactorial inheritance. With advances in genetic sequencing, evidence of familial clustering in VD has begun to highlight the genetic causes of these disorders, potentially opening up new avenues of treatment, particularly in Meniere's disease and disorders with comorbid hearing loss, such as Usher syndrome. In this review, we aim to present recent findings on the genetics of VDs, review the role of genetic sequencing tools, and explore the potential for individualized medicine in the treatment of these disorders.

**Methods:** A search of the PubMed database was performed for English language studies relevant to the genetic basis of and therapies for vestibular disorders, using search terms including but not limited to: “genetics,” “genomics,” “vestibular disorders,” “hearing loss with vestibular dysfunction,” “individualized medicine,” “genome-wide association studies,” “precision medicine,” and “Meniere's syndrome.”

**Results:** Increasing numbers of studies on vestibular disorder genetics have been published in recent years. Next-generation sequencing and new genetic tools are being utilized to unearth the significance of the genomic findings in terms of understanding disease etiology and clinical utility, with growing research interest being shown for individualized gene therapy for some disorders.

**Conclusions:** The genetic knowledge base for vestibular disorders is still in its infancy. Identifying the genetic causes of balance problems is imperative in our understanding of the biology of normal function of the vestibule and the disease etiology and process. There is an increasing effort to use new and efficient genetic sequencing tools to discover the genetic causes for these diseases, leading to the hope for precise and personalized treatment for these patients.

## Introduction

Vestibular disorders (VDs) are a heterogeneous group of conditions that stem from dysfunction of the inner ear, vestibulocochlear nerve, or central vestibular pathways. Patients with VDs typically present with vertigo, although other symptoms, including dizziness, unsteadiness, and oscillopsia, are often present as well. Benign inner ear conditions, including benign positional vertigo, Meniere's disease, and vestibular neuritis, are the common causes of vertigo. Other causes of isolated vestibular dysfunction include benign paroxysmal vertigo of childhood, bilateral vestibulopathy, and motion sickness. Due to similar developmental mechanisms between vestibular structures and the human hearing apparatus, some genetic hearing loss syndromes, such as Usher syndrome, also present with vestibular symptoms. Structural malformations of the inner ear, including enlarged vestibular aqueduct syndrome and superior canal dehiscence syndrome, are also causes of hearing and vestibular deficits (1). Vestibular migraine (VM), vestibular dysfunction occurring within the context of migraine headaches, is now also a well-established cause of vertigo (2).

There is growing evidence that genetics play a role in at least a subset of VDs (3). Multiple familial clusters with a range of vestibular symptoms have been described in the literature, and epidemiological studies have shown familial aggregation and higher prevalence of vestibular syndromes in some ethnic groups (4). However, the specifics of how genetic variations and familial clustering or aggregation may contribute to the development of specific VDs are largely unknown. This is due in part to limitations in clinical phenotyping, similarities in presentation of different VDs, and variable penetrance resulting in clinical heterogeneity (5).

Recently, advances in the field of genetic sequencing have increased our understanding about the pathophysiology of VDs. Genetic tools that have been used in genome-wide association studies in VDs include whole exome sequencing (WES) and whole genome sequencing (WGS). By using WES and WGS in combination with bioinformatic tools, novel VD gene variants have been identified, allowing for finer genetic differentiation between phenotypically similar conditions. As research into this area increases, it is important to realize the goals of this knowledge. As exemplified in the ever-evolving field of treatment for sensorineural hearing loss, genetic knowledge generated with these tools can lead to exciting and effective individualized gene therapies. In this review, we will examine new evidence of genetic contributions of VDs, use of genetic tools in the discovery of causal genes and individualized treatments, and future directions in VD treatment with a particular focus on the development of precision medicine (PM) platforms.

## Familial Meniere's Disease

Meniere's disease (MD) is a complex chronic inner ear disease; symptoms include episodic vertigo, fluctuating low-to-middle frequency SNHL, tinnitus, and aural fullness. The clinical course of MD is variable, but hearing loss is typically progressive. In 25–40% cases, hearing loss occurs bilaterally, though there is disagreement as to whether this represents a subtype of MD or a separate disorder (6). Per AAO-HNS criteria, a diagnosis of “definite MD” requires “2 or more episodes of vertigo lasting 20 min to 12 h, low-to-middle frequency hearing loss in one ear, fluctuating aural symptoms, and no other VD that better explains the symptoms” (7). More recently, [Lopez-Escamez et al. (6)] further divided MD into 5 clinical subtypes to better capture its clinical heterogeneity. Type 3, representing 13% of cases, is Familial MD (FMD), defined as patients meeting MD criteria with 2 or more 1st or 2nd degree relatives who also meet criteria (6).

FMD inheritance patterns have been widely studied, with most studies finding an autosomal dominant inheritance pattern; however, recessive and mitochondrial inheritance has been observed as well (8). This may indicate the existence of several different underlying genotypes that result in phenotypes meeting the clinical criteria of FMD. Several studies have published information on new genes associated with MD. Pathogenic variants in FAM136 and DTNA—both of which were found to code for proteins expressed in the vestibular neuroepithelium in adult rat models—were detected via WES in a single family with MD (9). In particular, a-Dystrobrevin—the protein product of DTNA—is associated with the dystrophin complex of proteins that is thought to play a role in cytoskeleton structure for cochlear hair cells, providing a potential mechanism for the SNHL seen in this family (9). Another family with multigenerational MD was found to have mutations in the PRKCB gene, which codes for a protein kinase and is expressed in both the adult human cochlea and semicircular canals (10). Notably, expression was greater in the tectal cells of the apical turn of the cochlea compared to the middle or basal turn, which may explain why SNHL tends to occur in lower frequencies in MD patients. Another study found pathogenic changes in DPT in one MD family, and in SEMA3D in another. Respectively coding for the extracellular matrix protein dermatopontin and the axonal guiding protein semaphoring-3D, both genes were found to be strongly expressed in the cochlea and the semicircular canals (11). Additionally, a family with a mutation in COCH, a gene related to the hearing loss disorder DFNA9, was found to have an MD-like phenotype with asymmetric SNHL, unilateral aural symptoms, and episodic vertigo (12). Recently, a rare variant analysis of FMD cases revealed the presence of missense variants in the *OTOG* gene (rs552304627) in ~1/3 of included families. This finding indicates that *OTOG* may represent a key gene in future genetic testing for FMD (13).

While the majority of MD cases are not familial, there is growing evidence of specific genotype-phenotype relationships underpinning the clinical presentation of sporadic MD. Mutations in MICA, TLR10, and NFKB1, all of which are associated with the immune system, have been associated with differences in the occurrence and rate of progression of SNHL in sporadic MD patients (14–16). Another recent study found that sporadic MD patients had enrichment of missense mutations in a variety of genes associated with SNHL including GJB2 (connexin 26 deafness), SLC26A4 (Pendred syndrome), and USH1G (Usher 1C) compared to controls, which the authors postulate may have an additive effect on the MD phenotype (17, 18).

### Vestibular Migraine

VM is a headache disorder with a prevalence of 1–3% that is characterized by episodes of migraine with vertigo (19). There is some evidence to suggest a heritable component to VM. A recent systematic review of VM prevalence studies indicated a moderate familial aggregation, with siblings of affected individuals at a four to 10 times greater risk than the general population (20). Symptoms of MD may also overlap with those of VM, making it difficult in some cases to distinguish between them clinically. Flook et al. (21) studied peripheral blood samples of patients with diagnosed MD and VM using WGS and cytokine panels and showed significant differences in gene expression and cytokine/chemokine profiles between the two patient populations. Further, two subgroups of MD were found, one with high IL-1β (MDH) and one with low IL-1β (MDL), supporting the theory that MD is a complex disorder with multiple endophenotypes. The authors proposed that a small cytokine assay of IL-1β, CCL3, CCL22, and CXCL1 levels would differentiate between VM and MD patients and may be used when a clinical diagnosis is unable to be made (21). MD as currently defined is a purely clinical diagnosis, yet is clinically heterogeneous, overlaps with other VDs, and has poorly understood pathophysiology. This state of affairs presents great challenges for both diagnosis and the development of effective treatments. The genetic discoveries summarized above can serve as a roadmap to more accurate, genotype-based classification of MD, and subsequently to better patient counseling and the development of new PM-based treatment approaches (22–24).

## Hereditary Sensorineural Hearing Loss With Vestibular Dysfunction

In addition to the isolated vestibulopathies, dizziness, and episodic vertigo is found in combination with moderate or severe hearing loss in a number of cochleovestibular disorders. Frequently, these disorders result from mutations in genes related to development of the otic capsule and temporal bone (5). These hereditary hearing disorders can be grouped by mode of inheritance into autosomal dominant, autosomal recessive, X-linked, or mitochondrial. Monogenic sensorineural hearing loss (SNHL) disorders that also present with vestibular dysfunction include DFNA9, DFNA11, DFNA15, and familial Meniere's disease. The mutations underlying these disorders are of interest to both hearing loss and vestibular disorder researchers and may benefit from precision medicine initiatives in both fields.

## DFNA

DFNA9 is an uncommon, delayed-onset disorder caused by heterozygous mutations in the COCH (coagulation factor C homology) gene (25). This disorder is primarily characterized by progressive high-frequency SNHL and can include vestibular dysfunction (gait imbalance, oscillopsia), ranging from bilateral vestibular loss to acute attacks resembling MD (12). Researchers have discovered 14 mutations in the COCH gene by studying unrelated families with DFNA9 (26). DFNA11 patients show a delayed-onset low-to-middle frequency hearing loss with a range of possible vestibular dysfunction. Seven mutations in the MYO7A gene have characterized as causes of DFNA11 (27). Myosin VIIA is a protein linked to hair bundle formation and mechanotransduction, and mutations in MYO7A are also related to Usher syndrome and DFNB2. Patients with DFNA15 display early-onset progressive high-frequency SNHL and vestibular dysfunction. DFNA15 is associated with missense mutations in the POU4F3 gene, which codes for a number of POU-domain transcription factors. Great variability in the type and severity of vestibular symptoms suggests that epigenetic factors or additional genes may be involved in defining the vestibular phenotype (28).

## Usher Syndromes

The Usher syndromes (USH) are a clinically and genetically diverse group of autosomal recessive disorders that result in dual hearing and visual loss along with vestibular dysfunction (29). Clinically, the Usher syndromes are divided into 3 groups (USH1, USH2, USH3) based on clinical presentation. Genetically, USH has been associated with 16 loci (9 USH1, 3 USH2, 2 USH3, 2 not specified), from which 13 individual genes have been identified (6 USH1, 3 USH2, 2 USH3, 1 modifier gene, 1 atypical USH gene) (30).

Patients with USH1—most commonly resulting from mutations in MYO7A (USH1B) which codes for the actin motor protein myosin VIIa—present with severe to profound hearing loss at birth as well as vestibular areflexia. Children with USH1 frequently are unable to walk until after 18 months of age and, as they grow, have difficulty with tasks requiring balance or coordination (31, 32).

Patients with USH2—most commonly resulting from mutations in USH2A (USH2A) which codes for the transmembrane protein usherin—are generally considered to have clinically normal vestibular function (33). However, in a study of 7 patients with genetically confirmed USH2 and without history of persistent imbalance or vestibular disorders, [Magliulo et al. (34)] found that the majority had pathologic responses on vestibular testing, pointing toward possible subclinical vestibular dysfunction in this patient population (34).

Patients with USH3—most commonly resulting from mutations in CLRN1 (USH3A) which codes for the transmembrane protein clarin-1—present with progressive vision and hearing loss occurring later in life than in patients with USH1 (35). Similarly, USH3 patients develop vestibular dysfunction less frequently and later in life. In a study of 22 USH3 patients with a median age of 30.5 years, Sadeghi et al. (36) found that 45% (10/22) had vestibular hypofunction or areflexia on caloric testing. Furthermore, 8 of the 10 affected patients had a normal walking age (<16 months), implying that their vestibular function was normal at that time and subsequently worsened over time (36).

While USH has been classically grouped into three USH types, in addition to atypical USH, recent genotype-phenotype correlation testing between USH1, USH2, and USH3 revealed that ~1/3 (29/90) of included patients displayed vestibular findings that were not consistent with their respective USH genotype (37). Wafa et al.'s findings suggest the absence of a reliable genotype-phenotype correlation in USH with respect to vestibular symptoms.

## Genetics of Hereditary Vestibular Disorders Without Hearing Loss

Genetic etiologies have been identified for several isolated vestibular disorders, such as benign paroxysmal vertigo of childhood and bilateral vestibulopathy. These disorders are often mistaken for vestibular migraine, with overlapping clinical presentation and concurrent diagnoses. With very few, if any, targeted interventions for these disorders, better understanding of the genetic underpinnings of these diseases may allow for both more accurate diagnoses and the development of more effective treatment modalities.

## Benign Paroxysmal Vertigo

Initially described as “benign recurrent vertigo” in a 1979 study of a group of patients with recurrent episodes of vertigo, benign paroxysmal vertigo (BPV) presents with recurrent vertiginous episodes lasting minutes to hours which first begin in childhood or early adulthood (38). Most patients with BPV eventually also meet International Headache Society (IHS) criteria for the diagnosis of migraine, which also displays a strong familial component. Furthermore, greater than 1/3 of first-degree relatives of BPV patients also suffer from BPV, and ½ of those relatives meet HIS criteria for migraine (39, 40). The largest genome wide study of BPV patients to date (20 families) showed evidence of linkage to chromosome 22q12 but also revealed significant genetic heterogeneity (24). Two other regions (5p15 and 3q24) also had NPL scores suggestive of linkage. Despite the strong association with migraine, linkage analysis of a broader phenotype of BPV or migraine headaches weakened the linkage signals compared to BPV alone. Therefore, there is no current evidence that isolated migraine is allelic with BPV (24). A more recent study of a three generation family with BPV linked the condition to chromosome 15, and found an autosomal dominant inheritance pattern (41).

## Familial Bilateral Vestibulopathy

Like benign paroxysmal vertigo of childhood, bilateral vestibulopathy (BVP) is characterized by recurrent vertigo attack. A significant portion of BVP patients suffer from migraine headaches as well (42); however the attacks are briefer in duration (ranging from seconds to minutes). Diagnostic criteria for BVP were proposed by the Bárány Society in 2017, requiring significant impairment of bilateral function of the vestibulo-ocular reflex (VOR)—seen in patients with “chronic unsteadiness when walking or standing—which worsen in darkness and/or on uneven ground, or during head motion.” For diagnosis, “horizontal angular VOR gain bilaterally should be <0.6 (angular velocity 150–300 degree/second), and/or the sum of the maximal peak velocities of the slow phase caloric-induced nystagmus for stimulation with warm and cold water <6 degree/second, and/or the horizontal angular VOR gain <0.1 upon sinusoidal stimulation on a rotatory chair and/or a phase lead >68 degree 9 time constant of <5 seconds).” Probable BVP is diagnosed with unsteadiness symptoms and bilateral pathologic bedside head impulse test (43). A small number of multiplex BVP families have been described, possibly because quantitative vestibular function testing is only available at major medical centers, making it challenging to identify families with bilateral vestibulopathy (44, 45). Unlike in familial deafness, where new genes have continued to be identified, no specific genes have been associated with BVP. One study was able to link BVP in four families to an area on chromosome 6q (45). However, a fifth family whose phenotype did not include migraines was not linked to 6q, suggesting that there may be multiple heterogeneous genotypes that meet the clinical diagnostic criteria. Given the relative rarity of the disease, large-scale efforts to identify and recruit patients with familial vestibulopathy are the next step to identify genes important to vestibular function that may underlie this disease (46).

## Hereditary Ataxias

Hereditary ataxias are a diverse group of inherited neurological disorders related to dysfunction of the cerebellum and brainstem and the associated afferent or efferent pathways. These disorders are monogenic and can be classified by pattern of inheritance as autosomal dominant, autosomal recessive, X-linked, or mitochondrial, with mutations having been identified in genes which code for components of ion channels. A subset of these disorders are associated with attacks of vertigo, likely due in part to vestibular connections within the cerebellum (47).

### Autosomal Dominant Ataxias

The autosomal dominant ataxias (ADAs) are a group of rare disorders with great genetic heterogeneity characterized by progressive ataxia, myoclonic epilepsy, dementia, and choreoathetosis. A number of ADAs are the result of point mutations, deletions or duplications associated with ion-channel dysfunction—spinocerebellar ataxias (SCA) 6, 13, 19/2, and episodic ataxias 1, 2, 5—or in signal transduction molecules (47). SCAs are a genetically diverse group characterized by slowly progressive ataxia. The clinical picture often overlaps between variations, making it difficult to diagnose from phenotype alone. However, genetic diagnosis has identified causative mutations in several SCA subtypes—which are numbered in chronological order of their causative locus or gene discovery– potentially opening the door to targeted treatment modalities (48–50). The most common ADA is SCA3 (Machado–Joseph disease), followed by SCA2, SCA1, and SCA6 (51). Episodic ataxias (EA), a group of classical monogenic recurrent vertigo syndromes, are early-onset, autosomal dominant neurologic disorders. Patients experience recurrent episodes of incoordination, slurred speech, and truncal ataxia. The EA subtypes are categorized by interictal findings and genetic mutations. Episodic ataxia type 1 (EA1) is characterized by short episodes of ataxia with interictal myokymia. EA1 is caused by mutations in *KCNA1* located on chromosome 12 (52), which encodes Kv1.1, a human homolog of the Shaker voltage-gated potassium channel in Drosophila (53). *KCNA1* is widely expressed in the cerebellum as well as along motor axons (54). The mutations causing EA1 result in compromised potassium channel activity, potentially leading to increased neuronal excitability (55, 56). EA2 patients suffer from episodes of vertigo with interictal nystagmus and progressive ataxia. Half of patients with EA2 have migraine headaches; EA2 shares several clinical symptoms with familial hemiplegic migraine type 1 (FHM1), basilar migraine, and progressive ataxia (57, 58). Both EA2 and FHM1 are caused by mutations in the *CACNA1A* gene, which codes for the a1A subunit of the P/Q-type voltage-gated calcium channel (59–61). Recently, exome sequencing has identified both novel mutations in *CACNA1C* and other genes that may be associated with EAs, such as *PRRT2* (62, 63). EA3, which has been documented in a single large Canadian family, is associated with episodic vertigo, nausea, tinnitus, ataxia, and migraine (64). While the disease locus has been mapped to chromosome 1q42, the causative gene is still unknown (65). EA4, also called familial periodic vestibulocerebellar ataxia, is characterized by episodic vertigo and ataxia beginning between ages 30 and 60 (66, 67). A whole genome scan failed to map the EA4 locus to a specific chromosomal location, making the designation of EA4 as a unique syndrome controversial. The causative gene is still undetermined. EA5 is characterized by episodic vertigo attacks beginning in the third decade of life, with episodes of vertigo and ataxia lasting several hours to days. EA5 was identified when several families with EA were screened and found to have mutations in the calcium channel b4 subunit CACNB4, on chromosome 2q. The associated gene encodes the b4 subunit of voltage-gated calcium channels and is the “most highly expressed β subunit in the cerebellum” (68). Interestingly, the EA5 mutation was discovered in a German family displaying generalized epilepsy without ataxia, raising questions about the variable expressivity of the mutation. EA6 was described in a child with episodic, progressive ataxia and migraine attacks, seizures, and prolonged alternating hemiplegia; MRI revealed edema in the corresponding hemisphere. A *de-novo* heterozygous mutation was detected from screening of the *SLC1A3* gene, a solute carrier gene that codes for excitatory amino acid transporter type 1 (EAAT1), a glial glutamate transporter in the cerebellum (69). EA7 was found in a single family with episodes of vertigo, weakness, dysarthria and ataxia for hours to days, with onset before age 20 (70). There are no interictal findings or tinnitus (which distinguishes it from EA3). Genome scanning mapped the locus of EA7 to chromosome 19q13 (70). However, the responsible gene has also not been definitively identified.

### Autosomal Recessive Ataxias

Autosomal recessive ataxias (ARAs) typically begin in childhood and are characterized by peripheral sensorimotor neuropathy. Friedreich ataxia and ataxia-telangiectasia are the most common ARAs (71). Next generation sequencing techniques (NGS) has defined new mutations in *GBA2* (72), ANO10 (73, 74), and *SYT14* genes (75); linkage analysis has identified new genes such as the gene *KIAA0226* (76). Both methods allow for a more precise diagnosis and prognosis, facilitating genetic counseling. Cerebellar ataxia, neuropathy, and vestibular areflexia syndrome (CANVAS), in contrast to the other autosomal recessive ataxias, is an adult-onset neurodegenerative disorder characterized by a spectrum of disease that can include sensory neuropathy, progressive unsteadiness, dizziness, and falls beginning around age 60. Cortese et al. recently published that biallelic intronic AAGGG repeat expansions in replication factor complex subunit 1 (*RFC1*) are the causative mutations in CANVAS. *RFC1* encodes a large subunit of the replication factor complex, which is a DNA-dependent ATPase that is essential for DNA replication and repair (77, 78).

### X-Linked Ataxias and Mitochondrial Ataxias

X-linked ataxias—occurring in males most commonly after age 50—are very rare, the most common being the fragile X-associated tremor/ataxia syndrome. The causative mutation is a 55–220 CGG repeat expansion in the fragile X mental retardation (PMR1) gene on chromosome Xq27.3, with expression profiling also showing deregulation in 14 microRNAs (79). Exome-sequencing has also identified a missense mutation in a kindred with X-linked with SCA (80). Very little is known of the genetics of mitochondrial ataxias, which are seen in diseases associated with cerebellar ataxia, such as Kearns-Sayre syndrome, myoclonic epilepsy with ragged-red fibers (MERRF), ataxia and retinitis pigmentosa (NARP), lactic acidosis and stroke-like episodes (MELAS), mitochondrial myopathy, neuropathy, and encephalopathy (80, 81).

## Discussion

### Current State of Personalized Treatment in VDs

Current treatment for vestibular disorders ranges from lifestyle modifications to surgical intervention. Initial treatment is directed toward identifying and reducing triggers for vestibular symptoms, which may vary even among family members. Medications used to treat vestibular symptoms include meclizine, dimenhydrinate, and acetazolamide, though these do not target underlying disease pathophysiology and can depress central compensatory mechanisms, leading to long-term worsening of symptoms. Meniere's disease can be treated with intratympanic injections of steroids or antibiotics, which have both been shown to be beneficial in symptom relief (82, 83). Ablative or destructive surgical procedures for Meniere's disease can lead to vertigo control but are associated with high risk of hearing loss (84). Given the lack of definitive treatment options, it is crucial to better understand the pathophysiology of VDs in order to develop more effective, individualized treatment approaches.

Precision medicine (PM) is a clinical approach that aims to prevent and treat disease on an individual basis, with consideration for individual variation in genetics, environments, and experiences. Success of PM rests on effective use of genetic tools, such as next generation sequencing (NGS) screening panels, targeted sequencing, and WGS and WES, both on an individual basis and also for the development of a genetic knowledge base. The potential inherent in a PM approach to disease can be seen in its use in the related field of hearing loss (HL), another condition with many heterogeneous genetic etiologies. Common HL-causing mutations are included on available NGS-based gene panels, which can be used for high-risk newborns and for large-scale population studies. WES has been used to find novel HL-associated genes and has aided homozygosity mapping in HL. Research approaches for personalized therapy for HL include gene therapy, stem cell therapy, and pre-implantation genetic diagnosis (85). The trajectory of PM in HL can serve as a road map for the application of PM to VDs.

While a comprehensive genetic knowledge base in VDs is in its relative infancy (Table 1), efforts to implement use of personalized medicine clinically in VDs are already under way, particularly in the diagnosis and treatment of MD. Genome England and the Meniere's Disease Consortium have created gene panels for sporadic (18) and familial MD (6); panelapp.com, which can potentially be used in large-scale genomic studies of MD. Further, [Lopez-Escamez et al. (6)] highlighted the multifactorial etiology of MD and proposed all individuals with MD symptoms obtain testing for rs4947296, a marker for a potentially treatable NF-kB-mediated inflammatory response and outlined an algorithm to determine candidacy for gene therapy (87). As more is discovered about the genetic bases of other VDs, similar algorithms can be generated and put into clinical practice.

**Table 1:** A summary of potential genes involved in vestibular disorders.

**Disorder**	**Gene or locus**	**References**
**Hereditary vestibular disorders without hearing loss**		
Benign Paroxysmal Vertigo of Childhood	22q12, 5p15, 3q24, chromosome 15	(24, 41)
Familial bilateral vestibulopathy	6q	(45)
**Hereditary Ataxias**		
CANVAS	*RFC1*	(77, 78)
Episodic ataxias	*KCNA1, CACNA1A*, 1q42, *CACNB4, SLC1A3*, 19q13	(52, 60, 65, 68–70)
Friedreich Ataxia	*FXN*	(71)
Spinocerebellar ataxias	*ATXN1, ATXN2, BEAN*	(48–51)
**Hereditary SNHL with vestibular dysfunction**		
DFNA9	*COCH*	(26)
DFNA11	*MYO7A*	(27)
DFNA15	*POU4F3*	(28)
Enlarged vestibular aqueduct syndrome	*SLC26A4, FOXI1, KCNJ10*	(1, 17, 86)
Familial Meniere Disease	*DTNA, FAM136, PRKCB, DPT, SEMA3D, COCH, OTOG*	(9–13)
Usher Syndrome	*MYO7A, USH2A, CLRN1*	(32–35)
**Episodic vestibular syndromes**		
Vestibular Migraine	5q35, 11q, 22q12	(22–24)

### Gene Expression Profiling, Gene Therapy, and Stem Cell Therapy Research in the Inner Ear

Multiple next-generation sequencing methods of gene expression profiling have been created, including microarray technology, serial analysis of gene expression (SAGE), and RNA-seq, and have led to the ability to study gene expression levels within the inner ear. Using microarray analysis, [Cristobal et al. (88)] assessed gene expression in vestibular epithelial cell types, eliciting more than 400 genes with differential expression between hair cells and supporting cells (88). Further, target genes showing specific expression in vestibular hair cells before and after maturation of mechano-sensitivity have also been defined, which could potentially serve in the future as gene therapy targets (89).

Many animal model studies of gene therapy in HL syndromes have included vestibular dysfunction studies. Recently, a number of studies have shown promise using gene therapy to restore auditory and vestibular function in mouse models of Usher syndrome (90–92). One study showed that the delivery of USH1c into the inner ear with an adeno-associated viral vector (Anc80L65) restored vestibular function—measured by rotarod performance and open field behavior—in USH1c mice nearly back to wild-type levels (92). Yet, to date, there have not been dedicated gene therapy studies targeting isolated vestibular symptoms in VDs. Similar to gene therapy, many stem cell therapy efforts for hearing loss syndromes include vestibular rescue as well. In addition, Taura et al. described a regenerative therapy for vestibular disorders utilizing human induced pluripotent stem cells (iPSCs) (93). Human neural stem cells (hNSCs) derived from iPSCs and injected into mouse utricle tissues produced elongated axon-like structures that contacted the vestibular hair cells. While the hNSCs showed signs of morphological maturation, they showed only partial physiological maturation, and further work must be done to investigate the potential of the therapy (93).

## Conclusions

Vestibular disorders are complex diseases, with heterogeneous presentations and overlapping symptoms making purely clinical diagnoses extremely challenging. Deep phenotyping with a complete familial medical history combined with NGS will allow for the identification of rare variants and genes related to familial vestibular disorders, allowing for more accurate classification of disease processes. In turn, furthering knowledge of genetic etiologies and pathophysiology of VDs will allow for the development and implementation of precision medicine approaches and individualized treatment to VDs, including gene and stem cell therapies.

## Author Contributions

CM, HD, EN, and TT: literature review, analysis of data, manuscript preparation, manuscript review, and manuscript submission. DY, AN, MS, YL, MH, SA, FT, GN, SB, and XL: manuscript preparation, manuscript review, and manuscript submission. All authors contributed to the article and approved the submitted version.

## Conflict of Interest

The authors declare that the research was conducted in the absence of any commercial or financial relationships that could be construed as a potential conflict of interest.

## References

[1] Yang T Vidarsson H Rodrigo-Blomqvist S Rosengren SS Enerback S Smith RJ. Transcriptional control of SLC26A4 is involved in Pendred syndrome and nonsyndromic enlargement of vestibular aqueduct (DFNB4). Am J Hum Genet. (2007) 80:1055–63. 10.1086/518314 17503324 PMC1867094

[2] Flint PW Haughey BH Robbins KT Thomas JR Niparko JK Lund VJ Cummings Otolaryngology - Head and Neck Surgery E-Book. Elsevier Health Sciences (2014). Available online at: https://books.google.com/books?id=lFajBQAAQBAJ

[3] Roman-Naranjo P Gallego-Martinez A Lopez Escamez JA Genetics of vestibular syndromes. Curr Opin Neurol. (2018) 31:105–10. 10.1097/WCO.0000000000000519 29095749

[4] Requena T Espinosa-Sanchez JM Lopez-Escamez JA. Genetics of dizziness: cerebellar and vestibular disorders. Curr Opin Neurol. (2014) 27:98–104. 10.1097/WCO.0000000000000053 24275721

[5] Frejo L Giegling I Teggi R Lopez-Escamez JA Rujescu D. Genetics of vestibular disorders: pathophysiological insights. J Neurol. (2016) 263(Suppl. 1):S45–53. 10.1007/s00415-015-7988-9 27083884 PMC4833787

[6] Lopez-Escamez JA Batuecas-Caletrio A Bisdorff A. Towards personalized medicine in Meniere's disease. F1000Res. (2018) 7:F1000 Faculty Rev-1295. 10.12688/f1000research.14417.1 30430003 PMC6097350

[7] Lopez-Escamez JA Carey J Chung WH Goebel JA Magnusson M Mandala M Diagnostic criteria for Meniere's disease. J Vestib Res. (2015) 25:1–7. 10.3233/VES-150549 25882471

[8] Requena T Espinosa-Sanchez JM Cabrera S Trinidad G Soto-Varela A Santos-Perez S. Familial clustering and genetic heterogeneity in Meniere's disease. Clin Genet. (2014) 85:245–52. 10.1111/cge.12150 23521103

[9] Requena T Cabrera S Martin-Sierra C Price SD Lysakowski A Lopez-Escamez JA. Identification of two novel mutations in FAM136A and DTNA genes in autosomal-dominant familial Meniere's disease. Hum Mol Genet. (2015) 24:1119–26. 10.1093/hmg/ddu524 25305078 PMC4834881

[10] Martin-Sierra C Requena T Frejo L Price SD Gallego-Martinez A Batuecas-Caletrio A. A novel missense variant in PRKCB segregates low-frequency hearing loss in an autosomal dominant family with Meniere's disease. Hum Mol Genet. (2016) 25:3407–15. 10.1093/hmg/ddw183 27329761 PMC5179939

[11] Martin-Sierra C Gallego-Martinez A Requena T Frejo L Batuecas-Caletrio A Lopez-Escamez JA. Variable expressivity and genetic heterogeneity involving DPT and SEMA3D genes in autosomal dominant familial Meniere's disease. Eur J Hum Genet. (2017) 25:200–7. 10.1038/ejhg.2016.154 27876815 PMC5255954

[12] Kim BJ Kim AR Han KH Rah YC Hyun J Ra BS. Distinct vestibular phenotypes in DFNA9 families with COCH variants. Eur Arch Otorhinolaryngol. (2016) 273:2993–3002. 10.1007/s00405-015-3885-1 26758463

[13] Roman-Naranjo P Gallego-Martinez A Soto-Varela A Aran I Moleon MDC Espinosa-Sanchez JM. Burden of rare variants in the OTOG gene in familial Meniere's disease. Ear Hear. (2020) 41:1598–605. 10.1097/AUD.0000000000000878 33136635

[14] Cabrera S Sanchez E Requena T Martinez-Bueno M Benitez J Perez N. Intronic variants in the NFKB1 gene may influence hearing forecast in patients with unilateral sensorineural hearing loss in Meniere's disease. PLoS ONE. (2014) 9:e112171. 10.1371/journal.pone.0112171 25397881 PMC4232390

[15] Gazquez I Moreno A Aran I Soto-Varela A Santos S Perez-Garrigues H. MICA-STR A.4 is associated with slower hearing loss progression in patients with Meniere's disease. Otol Neurotol. (2012) 33:223–9. 10.1097/MAO.0b013e31824296c8 22222578

[16] Requena T Gazquez I Moreno A Batuecas A Aran I Soto-Varela A. Allelic variants in TLR10 gene may influence bilateral affectation and clinical course of Meniere's disease. Immunogenetics. (2013) 65:345–55. 10.1007/s00251-013-0683-z 23370977

[17] Albert S Blons H Jonard L Feldmann D Chauvin P Loundon N. SLC26A4 gene is frequently involved in nonsyndromic hearing impairment with enlarged vestibular aqueduct in Caucasian populations. Eur J Hum Genet. (2006) 14:773–9. 10.1038/sj.ejhg.5201611 16570074

[18] Gallego-Martinez A Requena T Roman-Naranjo P Lopez-Escamez JA. Excess of rare missense variants in hearing loss genes in sporadic Meniere disease. Front Genet. (2019) 10:76. 10.3389/fgene.2019.00076 30828346 PMC6385525

[19] Li V McArdle H Trip SA Vestibular migraine. BMJ. (2019) 366:l4213 10.1136/bmj.l4213 31270067

[20] Paz-Tamayo A Perez-Carpena P Lopez-Escamez JA. Systematic review of prevalence studies and familial aggregation in vestibular migraine. Front Genet. (2020) 11:954. 10.3389/fgene.2020.00954 33110417 PMC7489493

[21] Flook M Frejo L Gallego-Martinez A Martin-Sanz E Rossi-Izquierdo M Amor-Dorado JC. Differential proinflammatory signature in vestibular migraine and meniere Disease. Front Immunol. (2019) 10:1229. 10.3389/fimmu.2019.01229 31214186 PMC6558181

[22] Bahmad F Jr DePalma SR Merchant SN Bezerra RL Oliveira CA Seidman CE. Locus for familial migrainous vertigo disease maps to chromosome 5q35. Ann Otol Rhinol Laryngol. (2009) 118:670–6. 10.1177/000348940911800912 19810609 PMC2767209

[23] Lee H Jen JC Cha YH Nelson SF Baloh RW. Phenotypic and genetic analysis of a large family with migraine-associated vertigo. Headache. (2008) 48:1460–7. 10.1111/j.1526-4610.2007.01002.x 18081823 PMC2846425

[24] Lee H Jen JC Wang H Chen Z Mamsa H Sabatti C. A genome-wide linkage scan of familial benign recurrent vertigo: linkage to 22q12 with evidence of heterogeneity. Hum Mol Genet. (2006) 15:251–8. 10.1093/hmg/ddi441 16330481

[25] Parzefall T Frohne A Koenighofer M Kirchnawy A Streubel B Schoefer C. Identification of a rare COCH mutation by whole-exome sequencing: implications for personalized therapeutic rehabilitation in an Austrian family with non-syndromic autosomal dominant late-onset hearing loss. Wien Klin Wochenschr. (2018) 130:299–306. 10.1007/s00508-017-1230-y 28733840 PMC5966484

[26] Robertson NG Cremers CW Huygen PL Ikezono T Krastins B Kremer H. Cochlin immunostaining of inner ear pathologic deposits and proteomic analysis in DFNA9 deafness and vestibular dysfunction. Hum Mol Genet. (2006) 15:1071–85. 10.1093/hmg/ddl022 16481359

[27] Sang Q Yan X Wang H Feng R Fei X Ma D. Identification and functional study of a new missense mutation in the motor head domain of myosin VIIA in a family with autosomal dominant hearing impairment (DFNA11). PLoS ONE. (2013) 8:e55178. 10.1371/journal.pone.0055178 23383098 PMC3558421

[28] van Drunen FJ Pauw RJ Collin RW Kremer H Huygen PL Cremers CW. Vestibular impairment in a Dutch DFNA15 family with an L289F mutation in POU4F3. Audiol Neurootol. (2009) 14:303–7. 10.1159/000212109 19372648

[29] Yan D Liu XZ. Genetics and pathological mechanisms of Usher syndrome. J Hum Genet. (2010) 55:327–35. 10.1038/jhg.2010.29 20379205 PMC4511090

[30] Mathur P Yang J. Usher syndrome: hearing loss, retinal degeneration and associated abnormalities. Biochim Biophys Acta. (2015) 1852:406–20. 10.1016/j.bbadis.2014.11.020 25481835 PMC4312720

[31] Koenekoop RK Arriaga MA Trzupek KM Lentz JJ Usher Syndrome Type I. In: Adam MP Ardinger HH Pagon RA Wallace SE Bean LJH Stephens K Amemiya A editors. GeneReviews((R)). (1993). Available online at: https://www.ncbi.nlm.nih.gov/pubmed/20301442 20301442

[32] Weil D Blanchard S Kaplan J Guilford P Gibson F Walsh J Defective myosin VIIA gene responsible for Usher syndrome type 1B. Nature. (1995) 374:60–1. 10.1038/374060a0 7870171

[33] Eudy JD Weston MD Yao S Hoover DM Rehm HL Ma-Edmonds M. Mutation of a gene encoding a protein with extracellular matrix motifs in Usher syndrome type IIa. Science. (1998) 280:1753–7. 10.1126/science.280.5370.1753 9624053

[34] Magliulo G Iannella G Gagliardi S Iozzo N Plateroti R Mariottini A. Usher's Syndrome type II: a comparative study of genetic mutations and vestibular system evaluation. Otolaryngol Head Neck Surg. (2017) 157:853–60. 10.1177/0194599817715235 28653555

[35] Joensuu T Hamalainen R Yuan B Johnson C Tegelberg S Gasparini P. Mutations in a novel gene with transmembrane domains underlie Usher syndrome type 3. Am J Hum Genet. (2001) 69:673–84. 10.1086/323610 11524702 PMC1226054

[36] Sadeghi M Cohn ES Kelly WJ Kimberling WJ Tranebjoerg L Moller C. Audiological findings in Usher syndrome types IIa and II (non-IIa). Int J Audiol. (2004) 43:136–43. 10.1080/14992020400050019 15198377

[37] Wafa TT Faridi R King KA Zalewski C Yousaf R Schultz JM. Vestibular phenotype-genotype correlation in a cohort of 90 patients with Usher syndrome. Clin Genet. (2020) 99:226–235. 10.1111/cge.13868 33089500 PMC7821283

[38] Slater R Benign recurrent vertigo. J Neurol Neurosurg Psychiatry. (1979) 42:363–7. 10.1136/jnnp.42.4.363 458483 PMC490208

[39] Lanzi G Balottin U Fazzi E Tagliasacchi M Manfrin M Mira E. Benign paroxysmal vertigo of childhood: a long-term follow-up. Cephalalgia. (1994) 14:458–60. 10.1046/j.1468-2982.1994.1406458.x 7697708

[40] Oh AK Lee H Jen JC Corona S Jacobson KM Baloh RW Familial benign recurrent vertigo. Am J Med Genet. (2001) 100:287–91. 10.1002/ajmg.1294 11343320

[41] Gizzi MS Peddareddygari LR Grewal RP. A familial form of benign paroxysmal positional vertigo maps to chromosome 15. Int J Neurosci. (2015) 125:593–6. 10.3109/00207454.2014.953157 25135283

[42] Baloh RW Jacobson K Fife T. Familial vestibulopathy: a new dominantly inherited syndrome. Neurology. (1994) 44:20–5. 10.1212/wnl.44.1.20 8290084

[43] Strupp M Kim JS Murofushi T Straumann D Jen JC Rosengren SM. Bilateral vestibulopathy: diagnostic criteria Consensus document of the Classification Committee of the Barany Society. J Vestib Res. (2017) 27:177–89. 10.3233/VES-170619 29081426 PMC9249284

[44] Brantberg K. Familial early-onset progressive vestibulopathy without hearing impairment. Acta Otolaryngol. (2003) 123:713–7. 10.1080/00016480310002500 12953770

[45] Jen JC Wang H Lee H Sabatti C Trent R Hannigan I. Suggestive linkage to chromosome 6q in families with bilateral vestibulopathy. Neurology. (2004) 63:2376–9. 10.1212/01.wnl.0000149498.79541.49 15623703

[46] Jen JC. Recent advances in the genetics of recurrent vertigo and vestibulopathy. Curr Opin Neurol. (2008) 21:3–7. 10.1097/WCO.0b013e3282f41ca0 18180645

[47] Hersheson J Haworth A Houlden H. The inherited ataxias: genetic heterogeneity, mutation databases, and future directions in research and clinical diagnostics. Hum Mutat. (2012) 33:1324–32. 10.1002/humu.22132 22689585

[48] Banfi S Servadio A Chung MY Kwiatkowski TJ Jr McCall AE Duvick LA. Identification and characterization of the gene causing type 1 spinocerebellar ataxia. Nat Genet. (1994) 7:513–20. 10.1038/ng0894-513 7951322

[49] Serrano-Munuera C Corral-Juan M Stevanin G San Nicolas H Roig C Corral J New subtype of spinocerebellar ataxia with altered vertical eye movements mapping to chromosome 1p32. JAMA Neurol. (2013) 70:764–71. 10.1001/jamaneurol.2013.2311 23700170

[50] Trott A Houenou LJ. Mini-review: spinocerebellar ataxias: an update of SCA genes. Recent Pat DNA Gene Seq. (2012) 6:115–21. 10.2174/187221512801327442 22670601

[51] Matilla-Duenas A. Machado-Joseph disease and other rare spinocerebellar ataxias. Adv Exp Med Biol. (2012) 724:172–88. 10.1007/978-1-4614-0653-2_14 22411243

[52] Browne DL Gancher ST Nutt JG Brunt ER Smith EA Kramer P. Episodic ataxia/myokymia syndrome is associated with point mutations in the human potassium channel gene, KCNA1. Nat Genet. (1994) 8:136–40. 10.1038/ng1094-136 7842011

[53] Papazian DM Schwarz TL Tempel BL Jan YN Jan LY. Cloning of genomic and complementary DNA from Shaker, a putative potassium channel gene from Drosophila. Science. (1987) 237:749–53. 10.1126/science.2441470 2441470

[54] Wang H Kunkel DD Martin TM Schwartzkroin PA Tempel BL. Heteromultimeric K+ channels in terminal and juxtaparanodal regions of neurons. Nature. (1993) 365:75–9. 10.1038/365075a0 8361541

[55] Adelman JP Bond CT Pessia M Maylie J. Episodic ataxia results from voltage-dependent potassium channels with altered functions. Neuron. (1995) 15:1449–54. 10.1016/0896-6273(95)90022-5 8845167

[56] Rea R Spauschus A Eunson LH Hanna MG Kullmann DM. Variable K(+) channel subunit dysfunction in inherited mutations of KCNA1. J Physiol. (2002) 538(Pt 1):5–23. 10.1113/jphysiol.2001.013242 11773313 PMC2290030

[57] Baloh RW Yue Q Furman JM Nelson SF. Familial episodic ataxia: clinical heterogeneity in four families linked to chromosome 19p. Ann Neurol. (1997) 41:8–16. 10.1002/ana.410410105 9005860

[58] Haan J Terwindt GM Ophoff RA Bos PL Frants RR Ferrari MD. Is familial hemiplegic migraine a hereditary form of basilar migraine? Cephalalgia. (1995) 15:477–81. 10.1046/j.1468-2982.1995.1506477.x 8706110

[59] Jun K Piedras-Renteria ES Smith SM Wheeler DB Lee SB Lee TG. Ablation of P/Q-type Ca(2+) channel currents, altered synaptic transmission, and progressive ataxia in mice lacking the alpha(1A)-subunit. Proc Natl Acad Sci USA. (1999) 96:15245–50. 10.1073/pnas.96.26.15245 10611370 PMC24805

[60] Mori Y Friedrich T Kim MS Mikami A Nakai J Ruth P. Primary structure and functional expression from complementary DNA of a brain calcium channel. Nature. (1991) 350:398–402. 10.1038/350398a0 1849233

[61] Ophoff RA Terwindt GM Vergouwe MN van Eijk R Oefner PJ Hoffman SM. Familial hemiplegic migraine and episodic ataxia type-2 are caused by mutations in the Ca^2+^ channel gene CACNL1A4. Cell. (1996) 87:543–52. 10.1016/s0092-8674(00)81373-2 8898206

[62] Gardiner AR Bhatia KP Stamelou M Dale RC Kurian MA Schneider SA. PRRT2 gene mutations: from paroxysmal dyskinesia to episodic ataxia and hemiplegic migraine. Neurology. (2012) 79:2115–21. 10.1212/WNL.0b013e3182752c5a 23077024 PMC3511930

[63] Hu Y Jiang H Wang Q Xie Z Pan S. Identification of a novel nonsense mutation p.Tyr1957Ter of CACNA1A in a Chinese family with episodic ataxia 2. PLoS ONE. (2013) 8:e56362. 10.1371/journal.pone.0056362 23441182 PMC3575407

[64] Steckley JL Ebers GC Cader MZ McLachlan RS. An autosomal dominant disorder with episodic ataxia, vertigo, and tinnitus. Neurology. (2001) 57:1499–502. 10.1212/wnl.57.8.1499 11673600

[65] Cader MZ Steckley JL Dyment DA McLachlan RS Ebers GC. A genome-wide screen and linkage mapping for a large pedigree with episodic ataxia. Neurology. (2005) 65:156–8. 10.1212/01.wnl.0000167186.05465.7c 16009908

[66] Damji KF Allingham RR Pollock SC Small K Lewis KE Stajich JM. Periodic vestibulocerebellar ataxia, an autosomal dominant ataxia with defective smooth pursuit, is genetically distinct from other autosomal dominant ataxias. Arch Neurol. (1996) 53:338–44. 10.1001/archneur.1996.00550040074016 8929156

[67] Farmer TW Mustian VM. Vestibulocerebellar ataxia. A newly defined hereditary syndrome with periodic manifestations. Arch Neurol. (1963) 8:471–80. 10.1001/archneur.1963.00460050021002 13944410

[68] Burgess DL Noebels JL. Voltage-dependent calcium channel mutations in neurological disease. Ann N Y Acad Sci. (1999) 868:199–212. 10.1111/j.1749-6632.1999.tb11287.x 10414295

[69] Jen JC Wan J Palos TP Howard BD Baloh RW. Mutation in the glutamate transporter EAAT1 causes episodic ataxia, hemiplegia, and seizures. Neurology. (2005) 65:529–34. 10.1212/01.wnl.0000172638.58172.5a 16116111

[70] Kerber KA Jen JC Lee H Nelson SF Baloh RW. A new episodic ataxia syndrome with linkage to chromosome 19q13. Arch Neurol. (2007) 64:749–52. 10.1001/archneur.64.5.749 17502476

[71] Montermini L Rodius F Pianese L Molto MD Cossee M Campuzano V. The Friedreich ataxia critical region spans a 150-kb interval on chromosome 9q13. Am J Hum Genet. (1995) 57:1061–7. 7485155 PMC1801369

[72] Hammer MB Eleuch-Fayache G Schottlaender LV Nehdi H Gibbs JR Arepalli SK. Mutations in GBA2 cause autosomal-recessive cerebellar ataxia with spasticity. Am J Hum Genet. (2013) 92:245–51. 10.1016/j.ajhg.2012.12.012 23332917 PMC3567281

[73] Maruyama H Morino H Miyamoto R Murakami N Hamano T Kawakami H. Exome sequencing reveals a novel ANO10 mutation in a Japanese patient with autosomal recessive spinocerebellar ataxia. Clin Genet. (2014) 85:296–7. 10.1111/cge.12140 23551081

[74] Vermeer S Hoischen A Meijer RP Gilissen C Neveling K Wieskamp N. Targeted next-generation sequencing of a 12.5 Mb homozygous region reveals ANO10 mutations in patients with autosomal-recessive cerebellar ataxia. Am J Hum Genet. (2010) 87:813–9. 10.1016/j.ajhg.2010.10.015 21092923 PMC2997370

[75] Doi H Yoshida K Yasuda T Fukuda M Fukuda Y Morita H. Exome sequencing reveals a homozygous SYT14 mutation in adult-onset, autosomal-recessive spinocerebellar ataxia with psychomotor retardation. Am J Hum Genet. (2011) 89:320–7. 10.1016/j.ajhg.2011.07.012 21835308 PMC3155161

[76] Alvarez-Mora MI Rodriguez-Revenga L Madrigal I Torres-Silva F Mateu-Huertas E Lizano E. MicroRNA expression profiling in blood from fragile X-associated tremor/ataxia syndrome patients. Genes Brain Behav. (2013) 12:595–603. 10.1111/gbb.12061 23790110

[77] Cortese A Simone R Sullivan R Vandrovcova J Tariq H Yau WY. Biallelic expansion of an intronic repeat in RFC1 is a common cause of late-onset ataxia. Nat Genet. (2019) 51:649–58. 10.1038/s41588-019-0372-4 30926972 PMC6709527

[78] Cortese A Tozza S Yau WY Rossi S Beecroft SJ Jaunmuktane Z. Cerebellar ataxia, neuropathy, vestibular areflexia syndrome due to RFC1 repeat expansion. Brain. (2020) 143:480–90. 10.1093/brain/awz418 32040566 PMC7009469

[79] Caramins M Colebatch JG Bainbridge MN Scherer SS Abrams CK Hackett EL. Exome sequencing identification of a GJB1 missense mutation in a kindred with X-linked spinocerebellar ataxia (SCA-X1). Hum Mol Genet. (2013) 22:4329–38. 10.1093/hmg/ddt282 23773993 PMC3792691

[80] Zeviani M Simonati A Bindoff LA Ataxia in mitochondrial disorders. Handb Clin Neurol. (2012) 103:359–72. 10.1016/B978-0-444-51892-7.00022-X 21827900

[81] Szmulewicz DJ Waterston JA MacDougall HG Mossman S Chancellor AM McLean CA. Cerebellar ataxia, neuropathy, vestibular areflexia syndrome (CANVAS): a review of the clinical features and video-oculographic diagnosis. Ann N Y Acad Sci. (2011) 1233:139–47. 10.1111/j.1749-6632.2011.06158.x 21950986

[82] Miller MW Agrawal Y Intratympanic Therapies for Meniere's disease. Curr Otorhinolaryngol Rep. (2014) 2:137–43. 10.1007/s40136-014-0055-8 25215266 PMC4157672

[83] Schoo DP Tan GX Ehrenburg MR Pross SE Ward BK Carey JP. Intratympanic (IT) Therapies for Meniere's Disease: Some Consensus Among the Confusion. Curr Otorhinolaryngol Rep. (2017) 5:132–41. 10.1007/s40136-017-0153-5 29568697 PMC5857942

[84] Volkenstein S Dazert S. Recent surgical options for vestibular vertigo. GMS Curr Top Otorhinolaryngol Head Neck Surg. (2017) 16:Doc01. 10.3205/cto000140 29279721 PMC5738932

[85] Rudman JR Mei C Bressler SE Blanton SH Liu XZ Precision medicine in hearing loss. J Genet Genomics. (2018) 45:99–109. 10.1016/j.jgg.2018.02.004 29500086

[86] Yang T Gurrola JG III Wu H Chiu SM Wangemann P Snyder PM. Mutations of KCNJ10 together with mutations of SLC26A4 cause digenic nonsyndromic hearing loss associated with enlarged vestibular aqueduct syndrome. Am J Hum Genet. (2009) 84:651–7. 10.1016/j.ajhg.2009.04.014 19426954 PMC2681005

[87] Gallego-Martinez A Espinosa-Sanchez JM Lopez-Escamez JA Genetic contribution to vestibular diseases. J Neurol. (2018) 265:29–34. 10.1007/s00415-018-8842-7 29582143

[88] Cristobal R Wackym PA Cioffi JA Erbe CB Roche JP Popper P. Assessment of differential gene expression in vestibular epithelial cell types using microarray analysis. Brain Res Mol Brain Res. (2005) 133:19–36. 10.1016/j.molbrainres.2004.10.001 15661362

[89] Schimmang T Maconochie M. Gene expression profiling of the inner ear. J Anat. (2016) 228:255–69. 10.1111/joa.12376 26403558 PMC4718171

[90] Emptoz A Michel V Lelli A Akil O Boutet de Monvel J Lahlou G. Local gene therapy durably restores vestibular function in a mouse model of Usher syndrome type 1G. Proc Natl Acad Sci USA. (2017) 114:9695–700. 10.1073/pnas.1708894114 28835534 PMC5594693

[91] Isgrig K Shteamer JW Belyantseva IA Drummond MC Fitzgerald TS Vijayakumar S. Gene therapy restores balance and auditory functions in a mouse model of Usher Syndrome. Mol Ther. (2017) 25:780–91. 10.1016/j.ymthe.2017.01.007 28254438 PMC5363211

[92] Pan B Askew C Galvin A Heman-Ackah S Asai Y Indzhykulian AA. Gene therapy restores auditory and vestibular function in a mouse model of Usher syndrome type 1c. Nat Biotechnol. (2017) 35:264–72. 10.1038/nbt.3801 28165476 PMC5340578

[93] Taura A Nakashima N Ohnishi H Nakagawa T Funabiki K Ito J. Regenerative therapy for vestibular disorders using human induced pluripotent stem cells (iPSCs): neural differentiation of human iPSC-derived neural stem cells after *in vitro* transplantation into mouse vestibular epithelia. Acta Otolaryngol. (2016) 136:999–1005. 10.1080/00016489.2016.1183169 27196942

